# Ligand-Dependent Associations of the Bovine TLR2 R152Q Variant with Neutrophil Oxidative Burst, Extracellular DNA Release, and Innate Immune Gene Expression

**DOI:** 10.3390/ijms27219578

**Published:** 2026-10-26

**Authors:** Joanna Sadowska, Dawid Tobolski, Zuzanna Polak, Milena Krupa, Roman Wójcik, Paweł Brym

**Affiliations:** 1 Department of Animal Genetics, Faculty of Animal Bioengineering, University of Warmia and Mazury in Olsztyn, Oczapowskiego 5, 10-719 Olsztyn, Poland; joanna.sadowska.3@doktorant.uwm.edu.pl; 2 Department of Large Animal Diseases and Clinic, Institute of Veterinary Medicine, Warsaw University of Life Sciences, 02-787 Warsaw, Poland; 3 Department of Epizootiology, Faculty of Veterinary Medicine, University of Warmia and Mazury in Olsztyn, Oczapowskiego 13, 10-718 Olsztyn, Poland; zuzanna.polak@uwm.edu.pl; 4 School of Veterinary Medicine, University College Dublin, Belfield, D04 V1W8 Dublin, Ireland; milena.krupa@ucdconnect.ie; 5 Department of Microbiology and Clinical Immunology, Faculty of Veterinary Medicine, University of Warmia and Mazury in Olsztyn, Oczapowskiego 13, 10-719 Olsztyn, Poland; brandy@uwm.edu.pl

**Keywords:** Toll-like receptor 2, R152Q polymorphism, bovine neutrophils, oxidative burst, extracellular dsDNA release, peptidoglycan, lipopolysaccharide, innate immunity gene expression

## Abstract

The bovine Toll-like receptor 2 (TLR2) R152Q substitution lies in an extracellular leucine-rich repeat domain and may alter pathogen-associated molecular pattern recognition, but its association with neutrophil function remains unknown. We genotyped 267 Polish Holstein–Friesian cows and evaluated isolated polymorphonuclear neutrophils (PMNs) after stimulation with the TLR2 agonist peptidoglycan (PGN) and lipopolysaccharide (LPS)–a well-documented PMN activator. The oxidative burst was assessed by the nitroblue tetrazolium reduction assay, extracellular double-stranded DNA release as an indirect index of neutrophil extracellular trap (NET)-associated DNA release by PicoGreen fluorescence, and the abundance of eight innate immune transcripts by RT-qPCR. Genotype frequencies were 0.59 for GG (coding for arginine), 0.39 for GA (heterozygotes), and 0.02 for AA (coding for glutamine), showing a significant deviation from Hardy–Weinberg equilibrium. In silico structural modeling predicted an allosteric destabilizing effect of the R152Q substitution. Functional responses of PMNs were both ligand- and concentration-dependent. The heterozygous GA PMNs exhibited the highest oxidative activity and extracellular dsDNA release following PGN stimulation. Conversely, AA PMNs demonstrated altered effector responses, including lower dsDNA release under LPS stimulation, accompanied by elevated basal transcript levels of recognition genes (*TLR2*, *TLR4*, *NCF1*, *PADI4*) but reduced *IRAK4* expression upon stimulation. These findings suggest that the TLR2 R152Q polymorphism is associated with ligand- and concentration-dependent variation in bovine PMN functional responses. Overall, the enhanced responses in GA PMNs point toward a potential heterozygote-associated functional advantage, whereas the predicted structural destabilization and altered effector responses in the rare AA genotype (*n* = 3) suggest possible selective constraints against mutant homozygotes, though these latter findings remain preliminary and require confirmation in larger cohorts.

## 1. Introduction

Innate immunity provides the first line of defense against invading pathogens. PMNs are major effector cells of innate immunity in cattle and rapidly migrate to sites of infection, where they use phagocytosis, degranulation, reactive oxygen species (ROS), and neutrophil extracellular traps (NETs) to restrict microbial spread [1,2,3,4]. These mechanisms contribute to bovine defense against economically important conditions, including mastitis, metritis, and parasitic infections [5,6,7,8,9,10].

Neutrophil activation begins with the recognition of pathogen-associated molecular patterns by pattern-recognition receptors, among which Toll-like receptors (TLRs) have a central role [11,12]. Bovine PMNs express multiple TLR proteins and respond functionally to TLR-associated stimuli [13,14]. Activation of these pathways coordinates oxidative activity, degranulation, cytokine expression, and extracellular DNA release.

Toll-like receptor 2 (TLR2) recognizes a broad spectrum of bacterial ligands, including lipoproteins, lipopeptides, and cell-wall components such as peptidoglycan (PGN) and related glycopeptides [15,16,17]. Structurally, PGN is an essential polymer composed of alternating N-acetylglucosamine and N-acetylmuramic acid residues cross-linked by peptide stems, forming a thick layer in Gram-positive bacteria and a thinner layer within the periplasmic space of Gram-negative bacteria [18]. Physiological cell-wall turnover continuously releases soluble muropeptides with immunologically active structures [19]. However, the role of TLR2 in the direct recognition of PGN remains controversial. Early studies identified PGN as one of the primary ligands for TLR2 [20,21]. Subsequent research suggested that the observed immunostimulatory activity might actually stem from co-purified contaminants, particularly lipoproteins and lipoteichoic acids (LTA) [22]. In addition, structural variations in PGN across different bacterial species, along with differences in isolation and purification protocols, further contribute to discrepancies observed between studies [18].

Nevertheless, commercially available PGN preparations with functionally validated TLR2 agonist activity serve as reliable tools for inducing inflammatory responses under experimental conditions [23,24]. By contrast, lipopolysaccharide (LPS)-the classical reference activator of PMNs-is recognized primarily through the TLR4 receptor complex [25]. At the intracellular level, ligand binding to TLR2 promotes its heterodimerization with TLR1 or TLR6 and the recruitment of the adaptor protein MyD88, interleukin-1 receptor-associated kinases (IRAKs), and tumor necrosis factor receptor-associated factor 6 (TRAF6), ultimately leading to the activation of the nuclear factor kappa B (NF-κB) and mitogen-activated protein kinase (MAPK) signaling pathways [26,27,28,29]. Because TLR4 shares key downstream signaling components with TLR2, potential crosstalk between these pathways can produce stimulus-dependent, nuanced functional outcomes in primary neutrophils [30].

TLR2-mediated recognition is highly relevant to bovine immune responses against various pathogens, including *Staphylococcus aureus*, *Streptococcus uberis*, *Mycoplasma bovis*, *Mycobacterium avium* subsp. *paratuberculosis*, and *Eimeria bovis* [31,32,33,34,35]. Host genetic variation can modify receptor structure, ligand binding, or downstream signal transduction, thereby contributing to individual differences in disease susceptibility or resilience. Accordingly, single-nucleotide variants (SNVs) in bovine *TLR2* have been widely investigated as potential genetic markers for disease resistance, with functional associations reported for paratuberculosis, bovine tuberculosis, and clinical mastitis [36,37,38,39,40].

However, the reported effects of these variants vary among populations, and the functional consequences of individual *TLR2* SNVs remain insufficiently characterized. Specifically, the R152Q variant (rs43706434; G>A) results in an arginine-to-glutamine substitution within the extracellular leucine-rich repeat (LRR) region of TLR2. Notably, this variant occurs at a notable frequency in Holstein–Friesian cattle in Poland, yet its functional impact on bovine neutrophils has not been established. To address this gap, this study investigated the association between the *TLR2* R152Q genotype and key neutrophil responses, specifically the oxidative burst, extracellular dsDNA release, and the expression of eight genes involved in microbial recognition and signaling, following stimulation with PGN-SA (*Staphylococcus aureus*), a functionally validated TLR2 agonist, or standard LPS as a reference activator.

## 2. Results

### 2.1. Genotyping of the TLR2 R152Q Variant by PCR-RFLP

**Figure 1:**
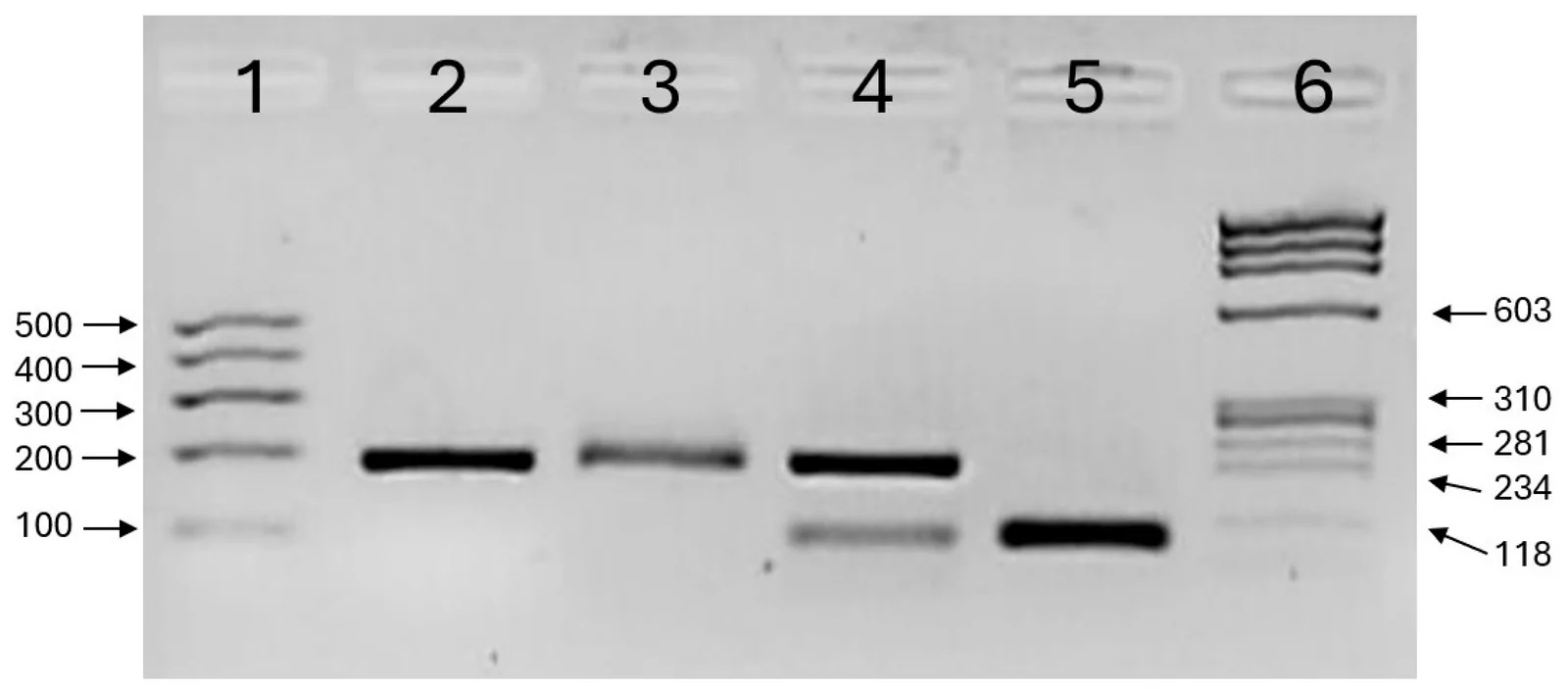
PCR-RFLP genotyping of the G>A transition (rs43706434) resulting in the R152Q variant in *TLR2* after *Pst*I digestion and separation on a 2.5% agarose gel. Lane 1: O’RangeRuler 100 bp DNA ladder; lane 2: undigested 194 bp PCR product; lane 3: GG genotype (194 bp); lane 4: GA genotype (194, 101, and 93 bp); lane 5: AA genotype (101 and 93 bp). The 93 and 101 bp fragments co-migrated and appeared as a single band; lane 6: ΦX174 DNA/*Hae*III marker.

A polymerase chain reaction–restriction fragment length polymorphism (PCR-RFLP) assay (Figure 1) was used to determine the TLR2 R152Q genotypes in 267 cows. The GG genotype, corresponding to arginine (R), was the most prevalent (0.59; *n* = 158), followed by the GA genotype (0.39; *n* = 104), whereas the AA genotype, encoding glutamine (Q), was rare (0.02; *n* = 5). The frequencies of the G and A alleles were 0.79 and 0.21, respectively (Table 1). The observed genotype distribution deviated significantly from Hardy–Weinberg equilibrium (χ^2^ = 6.87, df = 1, *p* < 0.01).

### 2.2. In Silico Analysis of the Predicted Structural Effect of R152Q in TLR2

According to the Project HOPE server [41], residue 152 is located within the extracellular leucine-rich repeat 5 (LRR5) domain of TLR2. The substitution of a positively charged, bulky arginine (R) with a neutral, smaller glutamine (Q) at position 152 alters local physicochemical properties, potentially compromising the local protein fold and function.

Further thermodynamic and conformational analysis using the bovine TLR2 structure model (AlphaFold DB: AF-Q95LA9-F1) via the DynaMut server [42] indicated a destabilizing effect of the R152Q variant. All underlying algorithms consistently predicted reduced overall structural stability (ΔΔ*G* = −0.513 kcal⋅mol^−1^) accompanied by increased molecular flexibility (ENCoM ΔΔ*S*_Vib_ = 0.287 kcal⋅mol^−1^⋅K^−1^).

**Figure 2:**
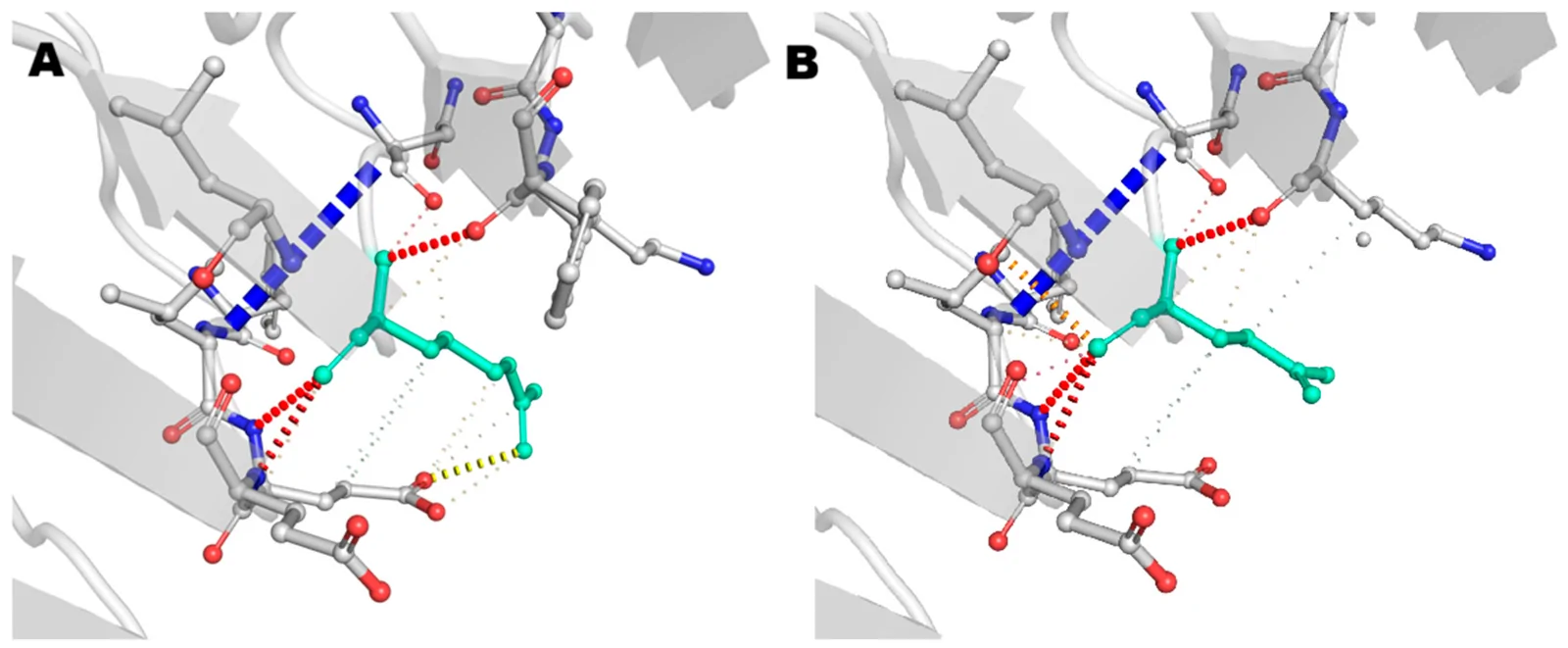
Predicted local interaction changes around residue 152 in the TLR2 R152Q variant. The wild-type arginine residue (**A**) and mutant glutamine residue (**B**) are highlighted in light blue. The model predicts alteration of the interaction network within the LRR domain, including loss of a potential ionic interaction (yellow dashed line). Hydrogen bonds are shown as red dashed lines and halogen bonds as blue dashed lines.

Mechanistically, the replacement of Arg152 disrupts local electrostatic interactions, most notably leading to the potential loss of a salt bridge with Glu177. These alterations may induce conformational shifts within the LRR repeats, ultimately disturbing the local structural organization and ligand-binding interface of TLR2. The predicted local interaction networks of the wild-type Arg152 and mutant Gln152 residues, including inter-residue contacts, are illustrated in Figure 2.

### 2.3. Association of R152Q with PMN Effector Activity

Functional assays were performed on 26 clinically healthy cows outside the periparturient period (GG, *n* = 11; GA, *n* = 12; AA, *n* = 3). The concentration of isolated PMNs ranged from 1.67 to 3.33 × 10^6^ cells/mL, and trypan blue exclusion indicated viability above 95%. Flow-cytometric quality control in eight randomly selected preparations showed a mean neutrophil proportion of 90.81% (SD = 6.2) and mean propidium iodide-negative viability of 98.2% (SD = 0.59). Early and late apoptotic cells accounted for 0.15% (SD = 0.07) and 1.04% (SD = 0.37), respectively. Isolated PMNs were stimulated with PGN-SA (10–60 µg/mL) or standard LPS (12.5–50 µg/mL). Oxidative activity was assessed using the nitroblue tetrazolium (NBT) reduction assay, whereas extracellular dsDNA release was quantified using PicoGreen fluorescence as a nonspecific index of NET-associated DNA release.

#### 2.3.1. Oxidative Activity

**Figure 3:**
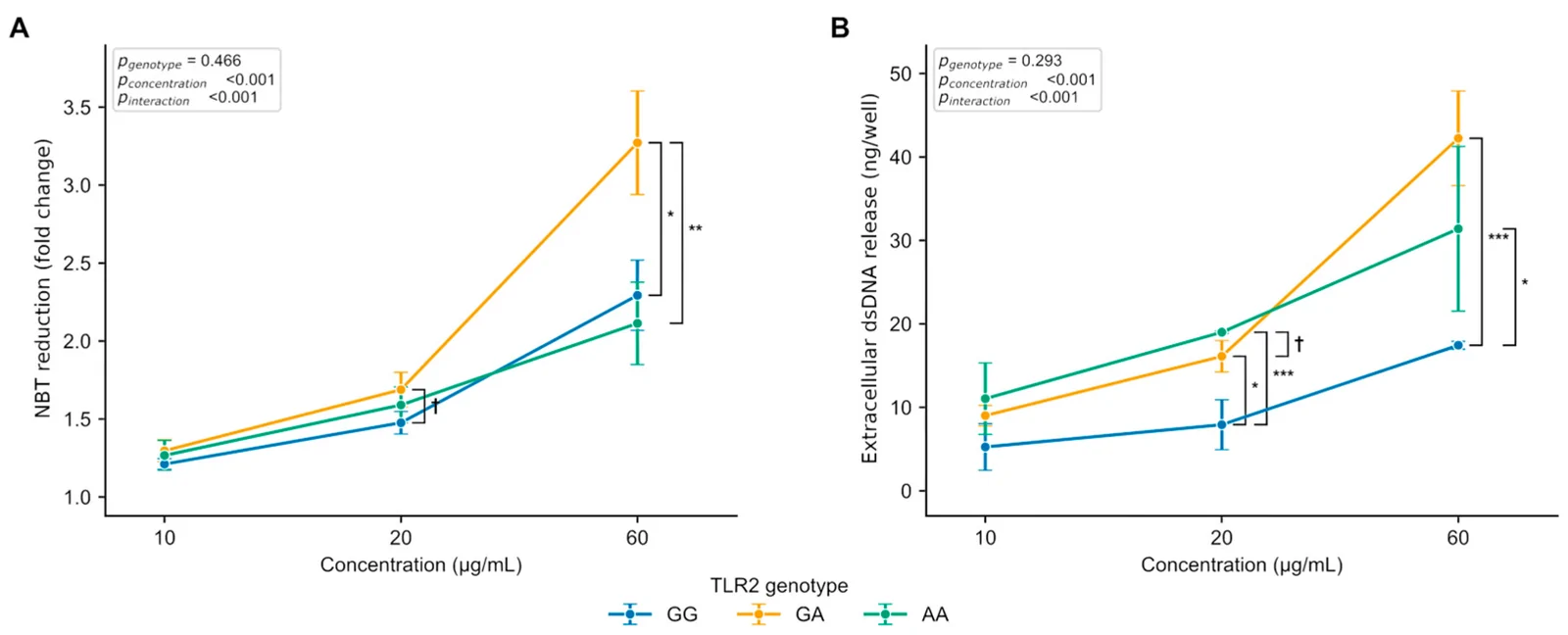
Association of the *TLR2* R152Q genotype with bovine polymorphonuclear neutrophil (PMN) effector responses after peptidoglycan (PGN) stimulation. (**A**) Nitroblue tetrazolium (NBT) reduction expressed as fold change relative to unstimulated control for each cow. (**B**) Extracellular double-stranded DNA (dsDNA) release, expressed as ng per well. Points show group means, and error bars show standard errors. Brackets denote unadjusted pairwise genotype contrasts within concentration. One, two, and three asterisks denote *p* < 0.05, *p* < 0.01, and *p* < 0.001, respectively, and † denotes *p* < 0.10. Panel (**B**) quantifies extracellular dsDNA release, a nonspecific index of neutrophil extracellular trap (NET)-associated DNA release.

PGN produces a concentration-dependent increase in NBT reduction (*p* < 0.001) with a genotype-by-concentration interaction (*p* < 0.001), while the overall genotype effect is not significant (*p* = 0.466, Figure 3A). Interestingly, heterozygous animals (GA) exhibited the highest NBT reduction. At 60 µg/mL PGN, GA PMNs show higher oxidative activity than GG (*p* < 0.05) and AA PMNs (*p* < 0.01).

**Figure 4:**
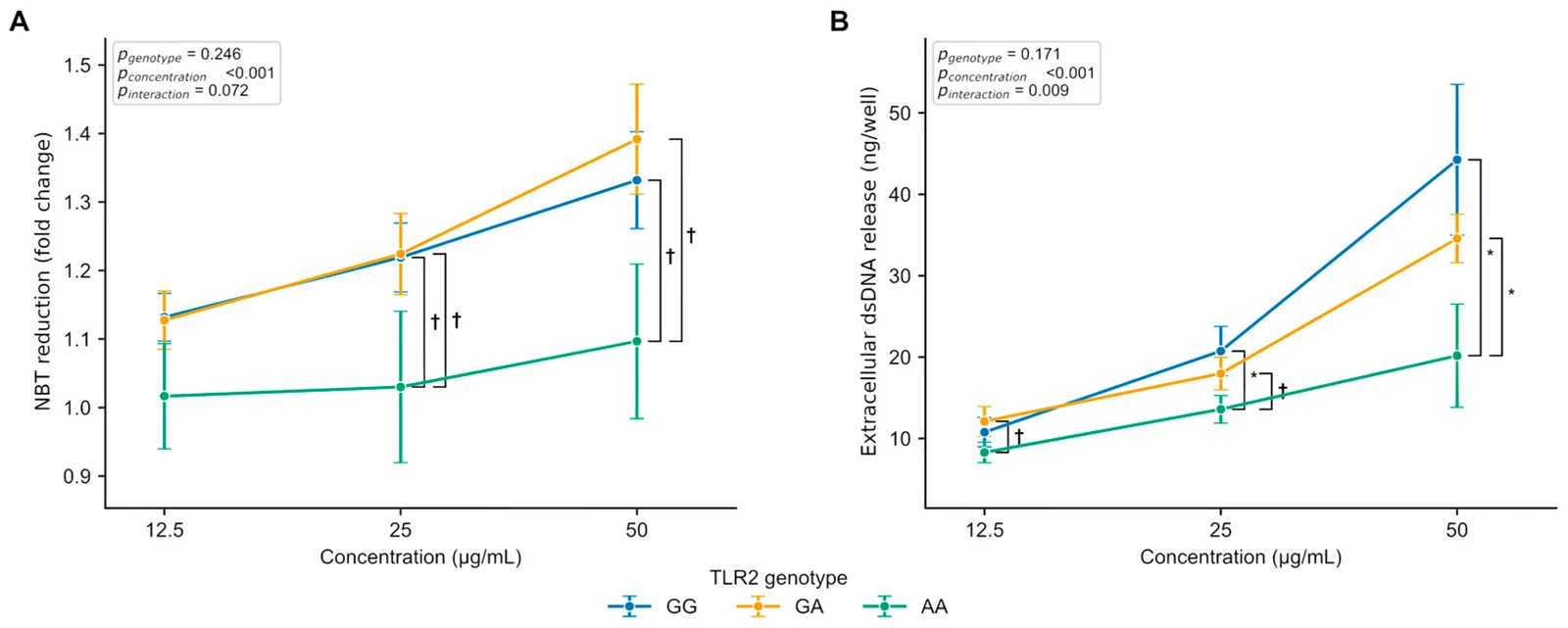
Association of the *TLR2* R152Q genotype with bovine polymorphonuclear neutrophil (PMN) effector responses after lipopolysaccharide (LPS) stimulation. (**A**) Nitroblue tetrazolium (NBT) reduction expressed as fold change relative to unstimulated control for each cow. (**B**) Extracellular double-stranded DNA (dsDNA) release, expressed as ng per well. Points show group means, and error bars show standard errors. Brackets denote unadjusted pairwise genotype contrasts within concentration. One asterisk denotes *p* < 0.05, and † denotes *p* < 0.10. Panel (**B**) quantifies extracellular dsDNA release, a nonspecific index of neutrophil extracellular trap (NET)-associated DNA release.

Additionally, stimulation with the reference stimulant LPS resulted in a concentration-dependent increase in NBT reduction (GEE concentration effect, *p* < 0.001). However, neither the overall genotype effect was significant (*p* = 0.246) nor did the genotype-by-concentration interaction reach statistical significance (*p* = 0.072, Figure 4A).

#### 2.3.2. Extracellular dsDNA Release

PGN-induced extracellular dsDNA release showed a concentration effect and a genotype-by-concentration interaction (both *p* < 0.001), whereas the overall genotype effect was not significant (*p* = 0.293, Figure 3B). At 20 µg/mL, PMNs with the GG genotype released lower amounts of extracellular dsDNA than those with the GA or AA genotype (*p* < 0.05). As observed for NBT reduction, at 60 µg/mL PGN, GA PMNs released significantly more extracellular dsDNA than either GG (*p* < 0.001).

Additionally, extracellular dsDNA release increases with LPS concentration (*p* < 0.001) and shows a genotype-by-concentration interaction (*p* = 0.009), whereas the overall genotype effect is not significant (*p* = 0.171, Figure 4B). At 50 µg/mL LPS, AA PMNs release less extracellular dsDNA than GG and GA PMNs (*p* < 0.05).

### 2.4. Expression of Genes Associated with PMN Recognition, Effector Function, and Signaling

**Figure 5:**
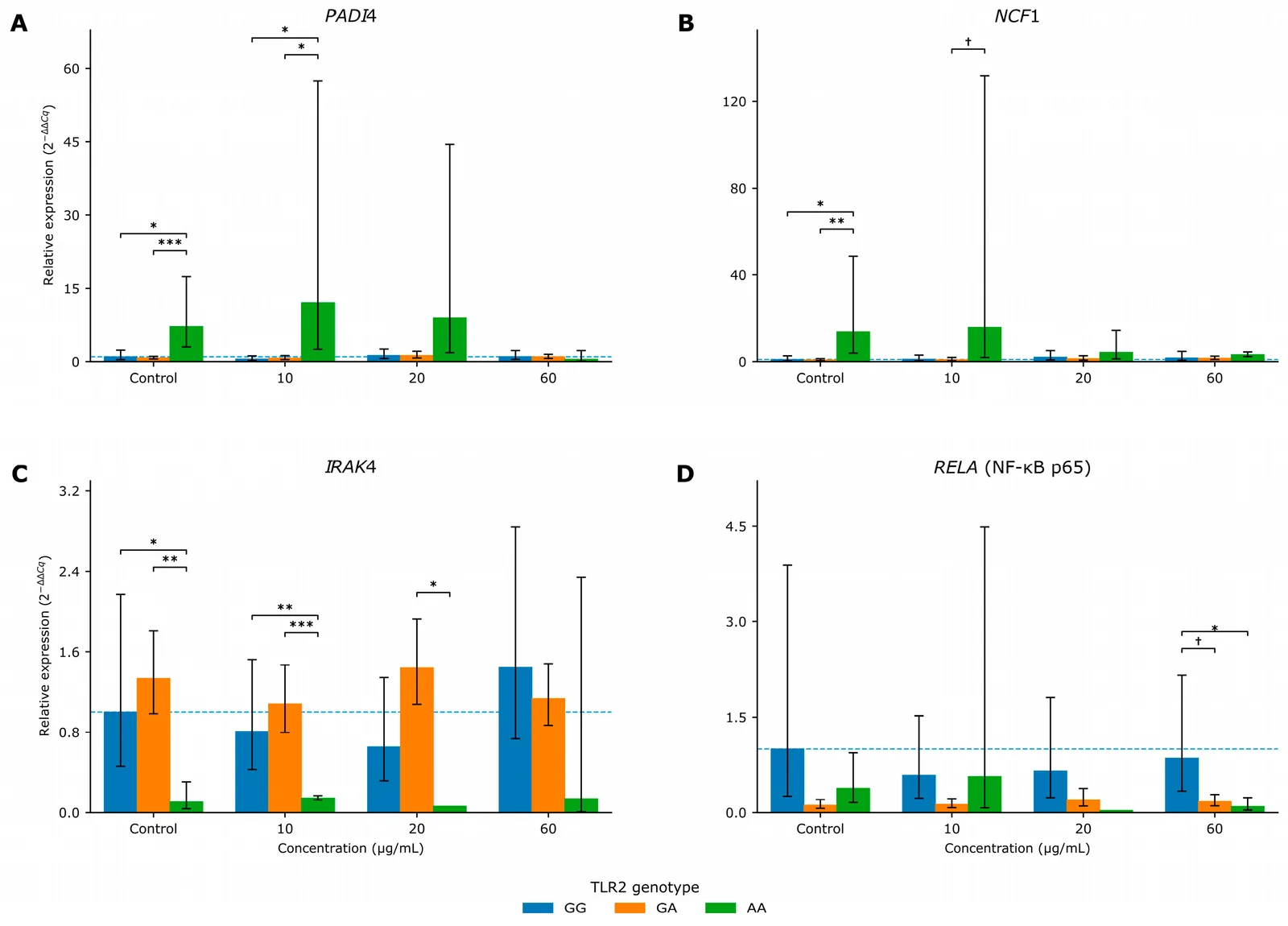
Relative transcript abundance after PGN stimulation for (**A**) *PADI4*, (**B**) *NCF1*, (**C**) *IRAK4*, and (**D**) *RELA* (NF-κB p65). Values were calibrated to the mean GG unstimulated control for each gene. Grouped bars show 2^(−mean ΔΔCq)^. Asymmetric error bars are the back-transformed mean ΔΔCq ± standard error, and the dashed horizontal line denotes relative expression of 1. Brackets show unadjusted genotype contrasts within concentration. One, two, and three asterisks denote *p* < 0.05, *p* < 0.01, and *p* < 0.001, respectively, and † denotes *p* < 0.10. Gene-specific y-axes are linear, begin at zero, and are scaled independently. Error bars are omitted when *n* = 1. The AA subsets for *IRAK4* and *RELA* at 20 µg/mL PGN each contain one observation.

**Figure 6:**
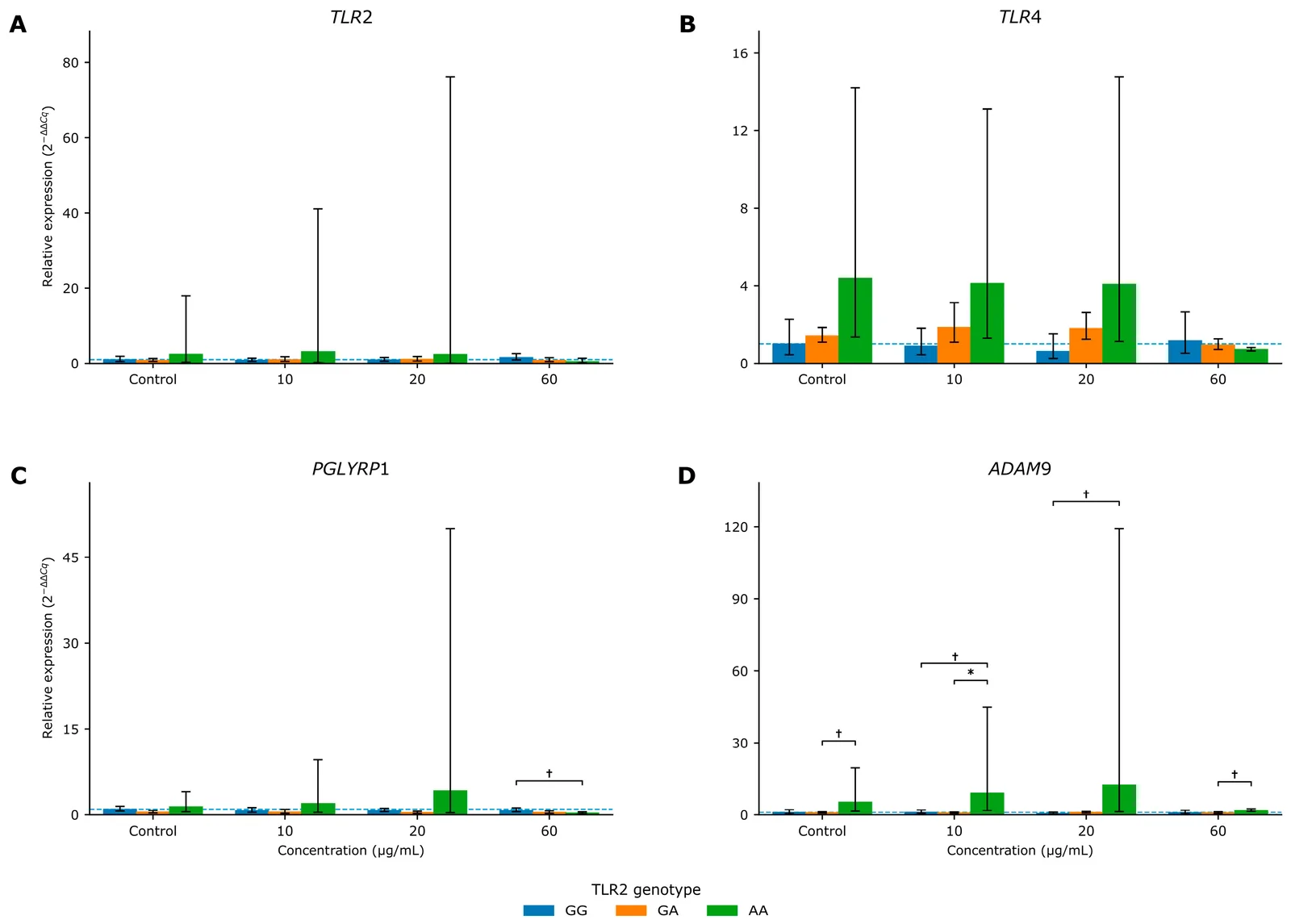
Relative transcript abundance after PGN stimulation for (**A**) *TLR2*, (**B**) *TLR4*, (**C**) *PGLYRP1*, and (**D**) *ADAM9*. Values were calibrated to the mean GG unstimulated control for each gene. Grouped bars show 2^(−mean ΔΔCq)^. Asymmetric error bars are the back-transformed mean ΔΔCq ± standard error, and the dashed horizontal line denotes relative expression of 1. Brackets show unadjusted genotype contrasts within concentration. One asterisk denotes *p* < 0.05, and † denotes *p* < 0.10. Gene-specific y-axes are linear, begin at zero, and are scaled independently. Error bars are omitted when *n* = 1.

RT-qPCR analysis included measurements from 27 cows (GG, *n* = 10, GA, *n* = 14, and AA, *n* = 3). Following PGN stimulation, PMNs from animals with the AA genotype showed higher transcript abundance of *PADI4*, *NCF1*, and *ADAM9* under selected conditions (Figure 5A,B and Figure 6D). Additionally, AA-genotype PMNs exhibited lower *IRAK4* and *RELA* mRNA expression compared with the other genotypes under selected conditions (Figure 5C,D). The R152Q variant was not associated with significant changes in *TLR2*, *TLR4*, or *PGLYRP1* expression following PGN stimulation (Figure 6A–C).

**Figure 7:**
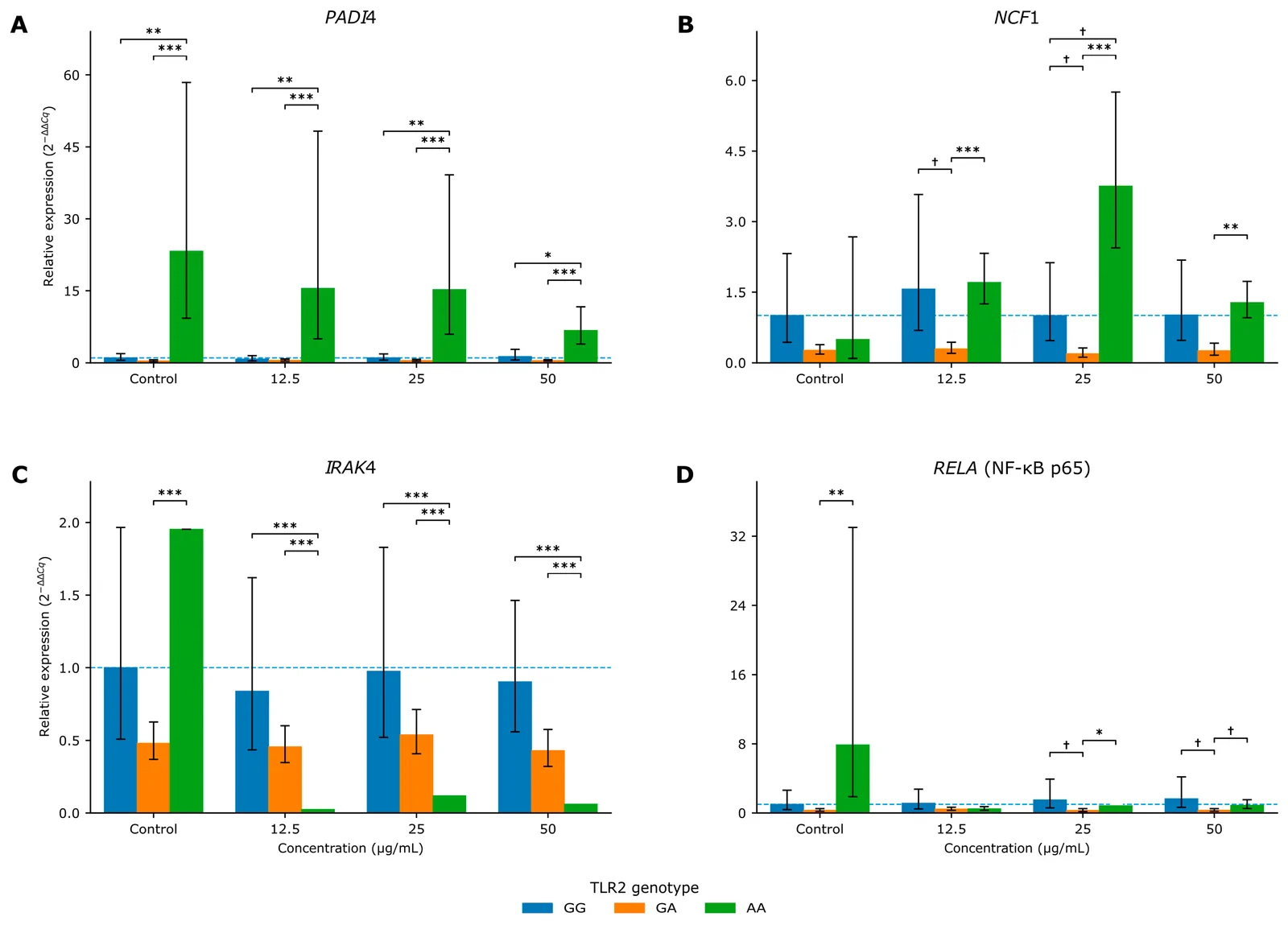
Relative transcript abundance after LPS stimulation for (**A**) *PADI4*, (**B**) *NCF1*, (**C**) *IRAK4*, and (**D**) *RELA* (NF-κB p65). Values were calibrated to the mean GG unstimulated control for each gene. Grouped bars show 2^(−mean ΔΔCq)^. Asymmetric error bars are the back-transformed mean ΔΔCq ± standard error, and the dashed horizontal line denotes relative expression of 1. Brackets show unadjusted genotype contrasts within concentration. One, two, and three asterisks denote *p* < 0.05, *p* < 0.01, and *p* < 0.001, respectively, and † denotes *p* < 0.10. Gene-specific y-axes are linear, begin at zero, and are scaled independently. Error bars are omitted when *n* = 1. The AA subsets for *IRAK4* at 12.5, 25, and 50 µg/mL LPS and for *RELA* at 25 µg/mL LPS each contain one observation.

**Figure 8:**
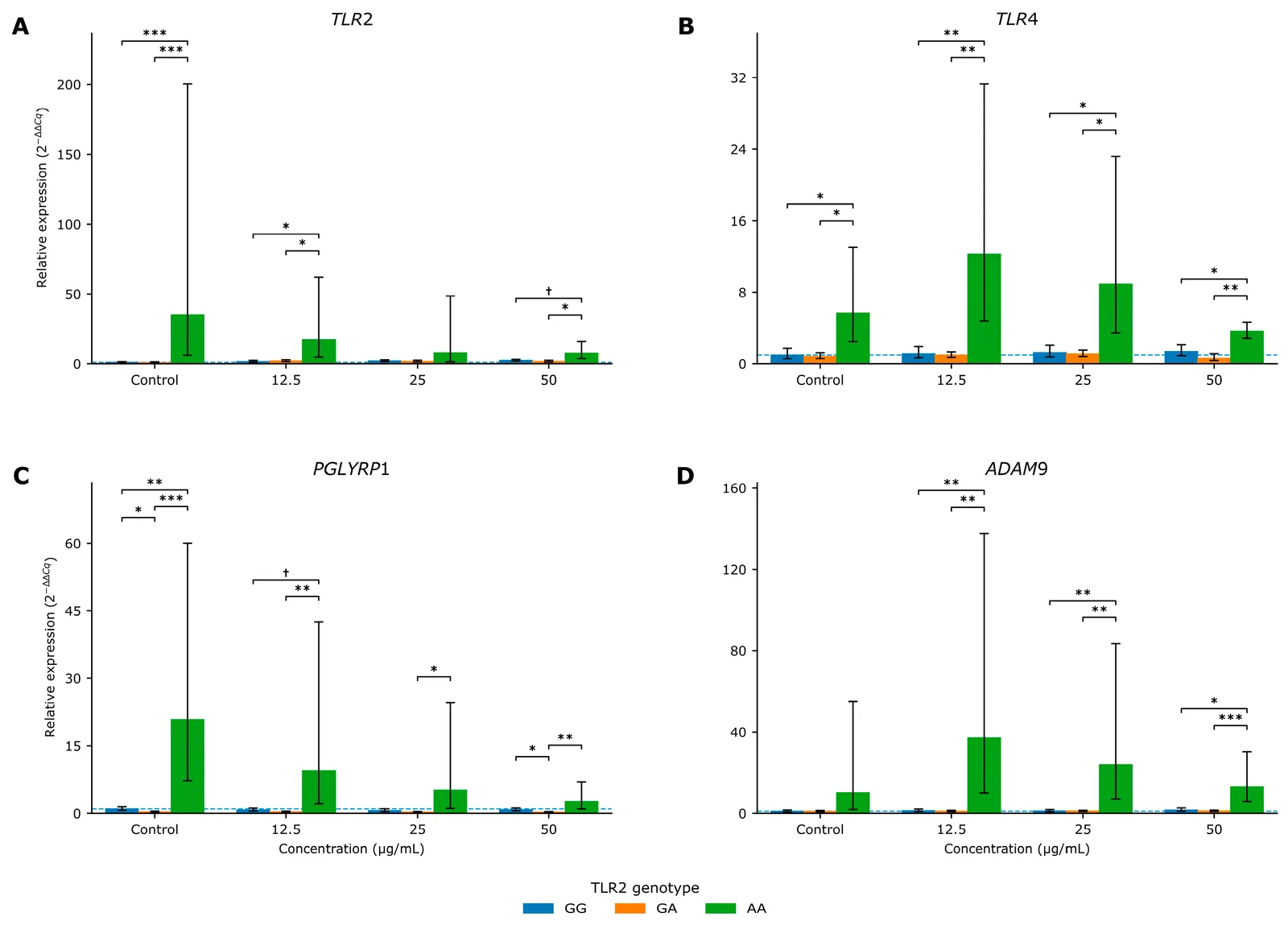
Relative transcript abundance after LPS stimulation for (**A**) *TLR2*, (**B**) *TLR4*, (**C**) *PGLYRP1*, and (**D**) *ADAM9*. Values were calibrated to the mean GG unstimulated control for each gene. Grouped bars show 2^(−mean ΔΔCq)^. Asymmetric error bars are the back-transformed mean ΔΔCq ± standard error, and the dashed horizontal line denotes relative expression of 1. Brackets show unadjusted genotype contrasts within concentration. One, two, and three asterisks denote *p* < 0.05, *p* < 0.01, and *p* < 0.001, respectively, and † denotes *p* < 0.10. Gene-specific y-axes are linear, begin at zero, and are scaled independently. Error bars are omitted when *n* = 1.

In response to LPS stimulation, PMNs from animals with the AA genotype showed higher transcript abundance of *PADI4*, *NCF1*, *TLR2*, *TLR4*, *PGLYRP1*, and *ADAM9* under selected conditions, although the pairwise genotype contrasts meeting the nominal significance threshold varied by concentration (Figure 7A,B and Figure 8). AA-genotype PMNs also showed lower *IRAK4* transcript abundance under selected conditions, and the *RELA* contrasts also varied by concentration (Figure 7C,D). These AA-associated patterns require cautious interpretation because of the small number of contributing cows.

## 3. Discussion

In this study, the R152Q polymorphism (rs43706434) in *TLR2* was evaluated in 267 Polish Holstein–Friesian cows. The GG genotype predominated (0.59), the GA genotype was frequent (0.39), and the AA genotype was rare (0.02). The observed genotype distribution showed a statistically significant deviation from Hardy–Weinberg equilibrium (HWE, *p* < 0.01), with an excess of heterozygotes and a deficit of AA homozygotes relative to HWE expectations. This departure describes the sampled herd, and while departures from HWE alone do not definitively identify underlying evolutionary causes, they provide an important signal of non-random population dynamics. Previous studies of bovine TLR polymorphisms [36,38,43] and evolutionary selection in TLR genes [44,45] provide a broader biological context for understanding how selective pressures shape innate immunity genes. Similarly, while associations of *TLR2* variants with somatic cell score or mastitis-related traits [46,47], candidate gene studies of milk production and mastitis [48,49], and analyses of bovine innate immune gene evolution [50] do not establish direct preferential culling or linkage in our specific herd, they outline plausible scenarios. Thus, while negative selection against AA homozygotes and heterozygote advantage cannot be proven from cross-sectional genotype data alone, they remain compelling working hypotheses that link our observations to broader population genetic concepts. Distinguishing these explanations would require multi-generation population and pedigree information together with genotype-specific disease, survival, and reproductive outcomes. Reports of low or undetected frequencies of this variant in other cattle populations [37,51] indicate a widespread pattern across breeds, although the exact population-genetic mechanisms remain to be fully elucidated.

The potential functional relevance of the R152Q substitution is further suggested by our in silico structural predictions. The substitution of a positively charged arginine with a neutral glutamine at position 152 is predicted to destabilize TLR2 and increase molecular flexibility, with the possible loss of an ionic interaction between Arg152 and Glu177. Although residue 152 in the LRR5 module lies outside the primary lipid-binding pocket and the TLR2/TLR1 heterodimerization interface described in structural studies [26], an allosteric effect is a plausible interpretation of the structural model. While comparative studies characterize the organization of TLR leucine-rich repeat domains [52,53], they do not directly demonstrate an R152Q-specific effect on folding or trafficking; nevertheless, changes in receptor stability, cell-surface abundance, ligand binding, and downstream signaling provide a strong rationale for the functional phenotypes observed here.

Previous studies of R152Q in cattle have yielded context-dependent findings. Koets et al. [33] reported that a *TLR2* haplotype containing the glutamine-encoding variant at position 152 was associated with greater NF-*κ*B pathway activity after stimulation with Pam3CSK4 and *Mycobacterium avium* subsp. *paratuberculosis*, as well as altered proinflammatory gene expression. By contrast, Ruiz-Larrañaga et al. [54] found no association between R152Q alone and infection status, although the A allele occurred within a haplotype associated with greater infection risk. These findings suggest that any association of R152Q may depend on haplotypic background, cell type, stimulus, and population, highlighting why single-variant effects require detailed phenotypic exploration rather than broad generalizations.

Unlike earlier work, centered mainly on monocytes or macrophages, this study evaluated PMN function. ROS production and NET formation are important neutrophil effector mechanisms [55,56,57]. The functional analysis of bovine PMNs after PGN stimulation identified significant associations between the R152Q genotype and selected PMN responses. PGN produced a concentration-dependent increase in NBT reduction, but significant genotype contrasts were confined to 60 µg/mL, where GA PMNs exceeded both homozygous groups. The individuals with AA genotype had the lowest numerical mean at that concentration, but it did not differ significantly from GG. The *NCF1* transcript pattern was not directly concordant with the NBT result. Although heterozygous animals exhibited significantly higher oxidative activity, this was not associated with increased *NCF1* mRNA expression. The highest basal *NCF1* transcript levels were observed in unstimulated PMNs with the AA genotype. However, *NCF1* expression declined with increasing PGN concentration and, at the highest stimulant concentration, did not differ significantly from that of the GG genotype. This lack of concordance between *NCF1* transcript abundance and oxidative activity is biologically plausible because activation of the NOX2 complex is regulated predominantly at the post-translational level, including phosphorylation and membrane translocation of cytosolic components such as p47phox (*NCF1*) and assembly of the active NADPH oxidase complex [11]. Therefore, *NCF1* mRNA abundance should not be expected to directly predict the magnitude of the acute oxidative burst.

Extracellular dsDNA release showed a different genotype-by-concentration pattern. At 60 µg/mL PGN, GA PMNs showed the highest mean extracellular dsDNA release. Because PicoGreen quantifies extracellular dsDNA irrespective of its cellular source, these data should be interpreted as a neutrophil-associated DNA release linked to cell activation rather than as an isolated, absolute measure of NET formation. Changes in *PADI4* transcript abundance may suggest altered transcriptional regulation of a pathway associated with chromatin modification, though transcript levels alone do not demonstrate direct PAD4 enzymatic activity or chromatin citrullination. The *PADI4* findings involving AA homozygotes require particular caution because of the small sample size (*n* = 3) and substantial variation among animals, meaning larger AA groups and protein-level measurements will be vital for future validation.

At 60 µg/mL PGN, GA PMNs showed robustly enhanced functional capacity (higher NBT reduction and peak extracellular dsDNA release). While these condition-specific responses do not establish a universal, lifelong fitness advantage in disease resistance, they offer a functional glimpse into potential heterozygote advantage. Differences in TLR2/TLR1 or TLR2/TLR6 complex function provide a compelling mechanistic framework: while the role of TLR2 coreceptors in ligand recognition is well established [19,58], expressing both allelic variants in heterozygous individuals could generate structurally and functionally diverse heterodimer populations. Although direct allele-specific receptor complex assembly was not measured here, this concept aligns with the broader evolutionary principle that maintaining immune-gene diversity can broaden host defense repertoires [59]. Thus, while the observed GA-associated responses do not constitute definitive proof of balancing selection in vivo, they strongly support further targeted investigation into how TLR2 structural variants fine-tune neutrophil reactivity.

Studies evaluating the effects of PGN stimulation on ROS production or NET release by bovine neutrophils remain limited. Nevertheless, studies of Gram-positive stimuli in bovine neutrophils have shown oxidative responses to *Staphylococcus aureus* [60,61,62] and NET-associated responses to the organism [6,63]. Xu et al. [23] reported impaired PGN-induced NET formation in *TLR2*- or *MyD88*-deficient murine neutrophils. Commercially available PGN is not necessarily a direct TLR2 agonist, and the TLR2 activity attributed to PGN preparations may result from copurified lipoproteins or other contaminants [19,22]. However, in the present study, we used PGN-SA from InvivoGen, a functionally validated TLR2 agonist.

LPS was used as a TLR4-associated neutrophil stimulus with documented effects on bovine ROS production and extracellular dsDNA release [64,65]. TLR4-associated and TLR2-associated pathways share *MyD88*- and *IRAK*-dependent components [11,66]. Nevertheless, because standard rather than ultrapure LPS was used, the presence of TLR2-active bacterial contaminants, including lipoproteins, cannot be completely excluded [67]. The use of both stimulants allowed us to investigate whether genotype-dependent differences were mainly related to TLR2-mediated responses or reflected broader alterations in shared signaling pathways and mechanisms regulating neutrophil effector functions.

LPS-associated responses also differed among R152Q genotypes. At 50 µg/mL LPS, AA PMNs released less extracellular dsDNA than GG or GA PMNs, whereas the overall genotype effect on NBT reduction was not significant. At the transcript level, AA PMNs often showed higher abundance of *PADI4*, *NCF1*, *TLR2*, *TLR4*, *PGLYRP1*, and *ADAM9*, but the significant contrasts varied by concentration. Importantly, while increased *TLR2* or *TLR4* transcript abundance should not be interpreted as definitive evidence of increased receptor availability at the neutrophil surface (as protein-level localization was beyond the scope of this assay), the coordinated upregulation of microbial recognition genes points toward a compensatory transcriptional adjustment under altered signaling conditions. In this context, AA-genotype PMNs also exhibited lower *IRAK4* transcript abundance following both PGN and LPS stimulation under several experimental conditions. *IRAK4* is a key component of the *MyD88*-dependent signaling pathway [68], and loss of *IRAK4* function impairs innate immune responses [69,70]. At the same time, TLR signaling pathways are tightly regulated by multiple feedback mechanisms [28,71]. Furthermore, the lack of direct correspondence between transcript abundance and neutrophil effector activity is well documented: mRNA abundance only partially reflects active protein levels, and cellular responses are heavily governed by translational and post-translational controls [72]. Therefore, although the observed *IRAK4* and *RELA* transcript patterns suggest altered regulation of downstream signaling, confirming changes in kinase activity, NF-*κ*B nuclear translocation, or pathway output will require targeted protein-level and phosphorylation analyses.

Overall, our findings suggest that the *TLR2* R152Q polymorphism is associated with notable, condition-dependent modulations of bovine neutrophil phenotypes. Structural predictions, transcript profiles, and functional readouts display complex patterns that highlight a sophisticated tuning of PMN effector mechanisms rather than a simplistic on/off switch.

Several limitations should be considered. The low frequency of the AA genotype in the evaluated herd naturally constrained the number of functional assays performed on AA homozygotes (*n* = 3), a common challenge when studying rare variants in commercial populations. Estimates involving AA homozygotes are therefore exploratory and must be interpreted with caution until confirmed in larger independent cohorts. Additionally, because broad cellular readouts (such as PicoGreen and NBT) reflect global activation states rather than isolated molecular events, and since *p* values were not adjusted for multiple comparisons, these findings should be treated as hypothesis-generating. Rather than diminishing the significance of our work, these complexities reveal the intricate regulatory networks governing innate immunity and establish the R152Q variant as a compelling candidate for future mechanistic research focused on protein-level signaling and direct receptor interactions.

## 4. Materials and Methods

### 4.1. Animals and Eligibility Criteria

Polish Holstein–Friesian dairy cows were enrolled from the teaching herd of the University of Warmia and Mazury in Olsztyn, Poland. Cows were housed in a free-stall barn, had continuous access to water, and received a total mixed ration twice daily. Animals remained under veterinary supervision, including daily monitoring of feed intake, rumination, fecal consistency, and general behavior. Functional and molecular phenotyping was performed after morning milking and outside physiological periods associated with pronounced periparturient immune and metabolic changes.

Eligibility was assessed using herd reproductive and lactation records and a physical examination. Inclusion criteria were the absence of clinical disease, no recorded chronic disorder, and a body condition score of 2.75–3.5. Udder examination and a rapid milk test for increased somatic cell count (Mastirapid; Vetoquinol, Lure, France) were performed on the sampling day. Cows with abnormal milk or rectal temperature > 39.5 °C were excluded. Hematology was evaluated using an Exigo Vet PWR analyzer (Boule Diagnostics AB, Spånga, Sweden) to screen for potential inflammatory conditions.

### 4.2. Blood Collection and Sample Allocation

Whole blood was collected from the coccygeal vein into vacuum tubes containing K_2_EDTA (Vacutainer; Becton Dickinson, Franklin Lakes, NJ, USA). For genotyping and hematology, 2 mL was collected from each cow. For PMN isolation, functional assays, and RNA-based analyses, 60 mL was collected. Samples were transported at 4 °C and processed within 1 h.

### 4.3. PCR-RFLP Genotyping

Genomic DNA was isolated from blood collected from 267 cows using the MasterPure Genomic DNA Purification Kit (Epicentre Biotechnologies, Madison, WI, USA) according to the manufacturer’s instructions. A 194 bp fragment encompassing rs43706434 was amplified with primers 5′-TGGTTCAGGTCCCTTTATGTCT-3′ and 5′-GCACTGATCTCAAGCTCCTC-3′. Amplicons were digested with FastDigest PstI (Thermo Fisher Scientific, Waltham, MA, USA), separated on a 2.5% agarose gel, and visualized under ultraviolet illumination.

### 4.4. In Silico Analysis of R152Q

The predicted structural consequences of R152Q were assessed with the HOPE web server [41]. Sequence annotation and residue numbering followed the bovine TLR2 UniProt entry Q95LA9 [73]. Protein stability and flexibility were evaluated with DynaMut [42] using the AlphaFold-predicted bovine TLR2 structure AF-Q95LA9-F1-v6 [74]. The predicted change in Gibbs free energy (ΔΔG) described stability, and the ENCoM change in vibrational entropy (ΔΔS_Vib_) described flexibility.

### 4.5. PMN Isolation and Quality Control

PMNs were isolated by density-gradient centrifugation with Ficoll-Paque PLUS (Cytiva, Uppsala, Sweden). Blood was diluted in 1× phosphate-buffered saline (PBS) containing 0.2 g/L EDTA, layered over Ficoll–Paque PLUS, and centrifuged at 450× *g* for 30 min at room temperature. Plasma and mononuclear cells were removed. The erythrocyte-granulocyte fraction underwent hypotonic lysis with sterile distilled water, after which isotonicity was restored with 2× PBS. Cells were washed and counted using a Neubauer hemocytometer. Viability was assessed by 0.04% trypan blue exclusion, and PMN purity was evaluated after May–Grünwald and Giemsa staining and by nuclear morphology after treatment with 2% acetic acid.

Flow cytometric quality control was performed for eight randomly selected preparations using a BD FACSAria II (Becton Dickinson, San Jose, CA, USA). Neutrophils were labeled with anti-granulocyte CH138A monoclonal antibody (1:100; Kingfisher Biotech, Saint Paul, MN, USA) and goat anti-mouse IgM Alexa Fluor 647 secondary antibody (1:420; Invitrogen, Waltham, MA, USA). Annexin V and propidium iodide staining (Roche, Basel, Switzerland) classified viable, early apoptotic, late apoptotic, and dead cells. A total of 10,000 events per sample were acquired at 100–300 events/s and analyzed with BD FACS Diva Software v6.1.3. Only preparations meeting the predefined purity and viability criteria were used.

### 4.6. NBT Reduction Assay

Oxidative burst was evaluated by NBT reduction (Sigma-Aldrich, St. Louis, MO, USA). PMNs were seeded at 1.25 × 10^6^ cells/well in 96-well plates and preincubated for 45 min in RPMI 1640 supplemented with 1% bovine serum albumin (BSA). Cells were stimulated with *Staphylococcus aureus* PGN at 10, 20, or 60 µg/mL (InvivoGen, San Diego, CA, USA; cat.code: tlrl-pgns2) or *Escherichia coli* O55:B5 LPS at 12.5, 25, or 50 µg/mL (Sigma-Aldrich, St. Louis, MO, USA; cat.code: L2880). PGN was used as a functionally validated TLR2 agonist, while LPS, a well-established TLR4 agonist, served as a reference stimulus. Concentration ranges were selected from published neutrophil stimulation studies [65,75]. NBT reduction proceeded for 40 min at 37 °C in 5% CO_2_. Formazan crystals were solubilized with 120 µL of 1 M potassium hydroxide (KOH; Aktyn, Poznań, Poland) and 140 µL of dimethyl sulfoxide (DMSO; Sigma-Aldrich, St. Louis, MO, USA). Absorbance was measured at 620 nm using a Multiskan FC microplate reader (Thermo Fisher Scientific, Waltham, MA, USA). NBT activity was expressed as fold change relative to the corresponding unstimulated control for each cow. Four technical replicates were analyzed per condition.

### 4.7. Extracellular dsDNA Quantification

Extracellular dsDNA was quantified using the Quant-iT PicoGreen dsDNA Assay Kit (Invitrogen, Carlsbad, CA, USA). PMNs were seeded at 5 × 10^5^ cells/well, preincubated for 45 min, and stimulated with LPS or PGN at the concentrations specified above. Cells were incubated for 2 h at 37 °C in 5% CO_2_. After centrifugation at 300× *g* for 5 min, 100 µL of cell-free supernatant was mixed with 100 µL of PicoGreen reagent in a black 96-well plate. Fluorescence was measured at 480 nm excitation and 520 nm emission using a Fluoroskan Ascent FL reader (Thermo Fisher Scientific, Waltham, MA, USA). Blank fluorescence was subtracted, and relative fluorescence units (RFU) were converted to dsDNA quantity using an eight-point standard curve (R^2^ > 0.99). Results were expressed as extracellular dsDNA per well (ng). Four technical replicates were analyzed per condition.

### 4.8. Short-Term In Vitro PMN Culture

For RNA-based analyses, isolated PMNs represented 27 cows (GG, *n* = 10, GA, *n* = 14 and AA, *n* = 3). PMNs were allocated to seven tubes, each containing 2 × 10^7^ cells in RPMI 1640 with 1% BSA, and preincubated for 45 min at 37 °C in 5% CO_2_. For each stimulus (PGN and LPS), an independent control aliquot of isolated PMNs was left unstimulated, while the remaining aliquots were treated with the corresponding three concentrations used in the functional assays. Because the PGN and LPS experiments were not always performed on the same day, the respective controls and stimulated aliquots were processed and assayed independently for each series. After 2 h, cells were lysed in TRIzol reagent (Thermo Fisher Scientific, Waltham, MA, USA), and lysates were stored at −80 °C.

### 4.9. RNA Isolation and cDNA Synthesis

Total RNA was extracted by the acid guanidinium thiocyanate–phenol–chloroform method [76] using TRIzol (Invitrogen, Thermo Fisher Scientific, Carlsbad, CA, USA) according to the manufacturer’s recommendations. RNA concentration and purity were assessed using a NanoDrop ND1000 spectrophotometer (Thermo Fisher Scientific, Wilmington, DE, USA). Samples were retained when A_260_/A_280_ was 1.9–2.1, A_260_/A_230_ was >1.8, and the RNA integrity number determined with an Agilent 2100 Bioanalyzer (Agilent Technologies, Waldbronn, Germany) and RNA 6000 Nano Kit was ≥7 (Agilent Technologies, Waldbronn, Germany).

Complementary DNA (cDNA) was synthesized from 1000 ng total RNA using the Transcriptor High Fidelity cDNA Synthesis Kit (Roche, Basel, Switzerland). Reactions (20 µL) contained oligo(dT)_18_ and random hexamer primers and were incubated at 25 °C for 10 min, 55 °C for 30 min, and 85 °C for 5 min. cDNA was diluted 1:5 in nuclease-free water and stored at −20 °C.

### 4.10. RT-qPCR

Transcript abundance of *TLR2*, *TLR4*, *PGLYRP1*, *ADAM9*, *PADI4*, *NCF1*, *IRAK4*, *RELA*, *RPLP0*, and *UCHL5* was measured using a LightCycler 480 II and SYBR Green I chemistry (Roche, Basel, Switzerland). Each 20 µL reaction contained 5 µL of five-fold diluted cDNA, 10 µL of LightCycler 480 SYBR Green I Master Mix, 10 pmol of each primer, and PCR-grade water. *RPLP0* and *UCHL5* were used jointly as internal reference genes, consistent with validated endogenous controls in bovine blood and neutrophil RT-qPCR [77,78]. Primers were designed with Primer3 [79] and are listed in Table 2.

Reactions were run in technical duplicate. No-template controls were run in triplicate. Cycling comprised 95 °C for 5 min and 45 cycles of 95 °C for 10 s, 60 °C for 15 s, and 72 °C for 20 s. Specificity was assessed using melting curves (78–89 °C; 0.11 °C/s) and 2.5% agarose gel electrophoresis. Gene-specific amplification efficiencies were evaluated using four-fold serial cDNA dilutions in triplicate. Quantification cycle (Cq) values were determined with LightCycler 480 Software v1.5 using the second-derivative maximum method.

### 4.11. Statistical Analysis

The cow was the biological replicate. Technical replicates were summarized within cow and condition. Genotype frequencies were compared with Hardy–Weinberg expectations using a chi-square test. For each stimulus, overall genotype, concentration, and genotype-by-concentration effects on NBT reduction and extracellular dsDNA were evaluated using Gaussian generalized estimating equations (GEE) with an exchangeable working correlation and cow as the cluster. Within each concentration, genotype effects were screened using Kruskal–Wallis tests, and pairwise comparisons were performed using Mann–Whitney *U* tests.

For RT-qPCR, the arithmetic mean of the *RPLP0* and *UCHL5* Cq values was subtracted from the target-gene Cq to obtain ΔCq. For each target gene and stimulus-specific analysis, the mean ΔCq of the GG unstimulated control group was the calibrator. Relative transcript abundance was calculated as 2^−ΔΔCq^ [80], while inference was performed on the ΔΔCq scale. Separate Gaussian GEE models with an exchangeable working correlation and cow-level clustering included TLR2 genotype, concentration, and their interaction. Treatment coding used GG and the unstimulated control as reference levels for term-level Wald tests. Pairwise genotype contrasts were calculated within each concentration. All reported *p* values were two-sided and unadjusted for multiple comparisons. Consequently, pairwise analyses were considered exploratory and should be interpreted with caution.

Relative-expression bar plots show back-transformed mean ΔΔCq values. Error-bar limits were calculated as 2^(−mean ΔΔCq ± SE)^. Standard errors were omitted for subsets containing only one biological observation. Specifically, for the AA genotype, only one valid biological observation was available for *IRAK4* and *RELA* at 20 µg/mL PGN, for *IRAK4* at 12.5, 25, and 50 µg/mL LPS, and for *RELA* at 25 µg/mL LPS. Missing measurements resulted from undetectable amplification in some samples or the exclusion of technical results that did not meet the quality-control criteria. Each gene was displayed on an independently scaled linear axis beginning at zero. Analyses were performed and Figure 3, Figure 4, Figure 5, Figure 6, Figure 7 and Figure 8 were generated using Python (version 3.15.2) with pandas (version 3.1.0), NumPy (version 2.5.4), Matplotlib (version 3.11.2), and statsmodels (version 0.15.0). Nominal statistical significance was defined as *p* < 0.05. The symbol † denotes unadjusted *p* < 0.10 in Figure 3, Figure 4, Figure 5, Figure 6, Figure 7 and Figure 8.

## 5. Conclusions

In conclusion, this study provides functional and structural evidence that the TLR2 R152Q polymorphism is associated with ligand- and concentration-dependent variation in bovine PMN functional responses. The enhanced effector capacity observed in GA PMNs (*n* = 12) compared to GG PMNs (*n* = 11) under PGN stimulation points toward a potential heterozygote advantage in bovine innate defense. While the predicted structural destabilization and altered functional patterns in the rare AA genotype suggest potential functional consequences of this mutation, the limited number of AA animals available for phenotyping (*n* = 3) precludes firm conclusions regarding selective mechanisms. These preliminary findings regarding the mutant homozygote require confirmation in larger, independent cattle populations.

## Figures and Tables

**Table 1:** Genotype and allele frequencies for *TLR2* R152Q (rs43706434) in 267 Polish Holstein–Friesian cows.

Genotype	*n*	Frequency	Allele	Frequency
GG	158	0.59	G	0.79
GA	104	0.39	A	0.21
AA	5	0.02		

**Table 2:** Primer sequences and RT-qPCR amplicon characteristics. Sequences are presented in the 5′–3′ orientation. The annealing temperature was 60 °C for all primer pairs.

Gene Symbol	Gene Name	Primer Sequence (5′–3′)	Product Tm (°C)	Amplicon (bp)
*TLR4*	Toll-like receptor 4	F: TGGAGCTCTATCGCCTTCTG R: TTCTTGCGGGTTGGTTTCTG	81.10	158
*ADAM9*	A disintegrin and metalloproteinase 9	F: TGCTGCAGAAGGAAGAGATCACAGA R: CTGAGGCTGCCTGGCTCCGTAA	82.16	174
*PGLYRP1*	Peptidoglycan recognition protein 1	F: GCCCAAAACGTGCAGTACTA R: GAGCCCATCTTCTCCGATCA	82.65	84
*TLR2*	Toll-like receptor 2	F: TTGCTCCTGTGACTTCCTGT R: GAGAGTCACAGCGGTAGTCA	78.07	92
*RELA*	RELA proto-oncogene, NF-κB subunit	F: ATCTTTGACAACCGCGCCCCC R: TGCCGGTGCACGTCAGCTTG	87.43	194
*IRAK4*	Interleukin-1 receptor-associated kinase 4	F: GCTTCGGTTTCTGCTGAACT R: CCCCACGTGTGAGTTACTG	81.51	185
*PADI4*	Peptidyl arginine deiminase 4	F: CCTCACTGGGGTCGAAATCT R: TTGGGGTTGTCTCTGTCACA	87.85	153
*NCF1*	Neutrophil cytosolic factor 1	F: CGCTTCACCGAGATCTACGA R: ATCTTGACAGGCAGGCTCAT	89.05	197
*RPLP0*	Ribosomal protein lateral stalk subunit P0	F: CAACCCTGAAGTGCTTGACAT R: AGGCAGATGGATCAGCCA	86.14	227
*UCHL5*	Ubiquitin C-terminal hydrolase L5	F: ACAAAGACAACTTGCTGAGGAACCC R: GGCAACCTCTGACTGAATAGCACTT	81.16	79

F, forward primer; R, reverse primer; Tm, melting temperature.

## Data Availability

The datasets used and/or analysed during the current study are available from the corresponding author on reasonable request.

## Abbreviations

The following abbreviations are used in this manuscript:

BSA	bovine serum albumin
cDNA	complementary DNA
Cq	quantification cycle
DMSO	dimethyl sulfoxide
dsDNA	double-stranded DNA
EDTA	ethylenediaminetetraacetic acid
ENCoM	elastic network contact model
GEE	generalized estimating equations
IRAK	interleukin-1 receptor-associated kinase
KOH	potassium hydroxide
LPS	Lipopolysaccharide
LRR	leucine-rich repeat
NBT	nitroblue tetrazolium
NET	neutrophil extracellular trap
NF-κB	nuclear factor kappa B
PBS	phosphate-buffered saline
PCR-RFLP	polymerase chain reaction–restriction fragment length polymorphism
PGN	Peptidoglycan
PMN	polymorphonuclear neutrophil
RFU	relative fluorescence unit
ROS	reactive oxygen species
RT-qPCR	reverse-transcription quantitative polymerase chain reaction
